# Truncated WT1 Protein Isoform Expression Is Increased in MCF-7 Cells with Long-Term Estrogen Depletion

**DOI:** 10.1155/2021/6282514

**Published:** 2021-11-20

**Authors:** Saavedra-Alonso Santiago, Zapata-Benavides Pablo, Mendoza-Gamboa Edgar, Chavez-Escamilla Ana Karina, Arellano-Rodríguez Mariela, Rodriguez-Padilla Cristina

**Affiliations:** Department of Immunology and Virology, Faculty of Biological Sciences, Autonomous University of Nuevo Leon (UANL), San Nicolas de los Garza, Nuevo Leon 66450, Mexico

## Abstract

**Background:**

The *wt1* gene codes for a transcription factor that presents several protein isoforms with diverse biological properties, capable of positively and negatively regulating genes involved in proliferation, differentiation, and apoptosis. WT1 protein is overexpressed in more than 90% of breast cancer; however, its role during tumor progression is still unknown. *Methodology*. In this work, we analyzed the expression of WT1 isoforms in several breast cancer cells with different tumor marker statuses and an *in vitro* assay using MCF-7 cells cultured with long-term estrogen depletion (MCF-7 LTED cells) with the finality to mimic the process of switching from hormone-dependent to hormone-independent. Moreover, growth kinetics, sensitivity to tamoxifen, and relative expression analysis of ER and Her2/neu were performed.

**Results:**

Initially, the expression of 52-54 kDa protein isoform of WT1 in the breast cancer cell line ER (+) was detected by western blot and was absent in ER (-), and the 36-38 kDa protein isoform of WT1 was detected in all cell lines analyzed. The analysis of alternative splicing by RT-PCR shows that the 17AA (+)/KTS (-) isoform of WT1 was the most frequent in the four cell lines analyzed. *In vitro*, the MCF-7 cells in the estrogen depletion assay show an increase in the expression of the 52-54 kDa isoform of WT1 in the first 48 hours, and this was maintained until week 13, and later, this expression was decreased, and the 36-38 kDa isoform of WT1 did not show change during the first 48 hours but from week 1 showed an increase of expression, and this remained until week 27. Growth kinetic analysis showed that MCF-7 LTED cells presented a 1.4-fold decrease in cellular proliferation compared to MCF-7 cells cultured under normal conditions. In addition, MCF-7 LTED cells showed a decrease in sensitivity to the antiproliferative effect of tamoxifen (*p* ≤ 0.05). Samples collected until week 57 analyzed by qRT-PCR showed an increase in the relative expression of the Her2/neu and ER.

**Conclusions:**

Modulation of protein isoforms showed differential expression of WT1 isoforms dependent on estrogen receptor. The absence of 52-54 kDa and the presence of the 36-38 kDa protein isoform of WT1 were detected in ER-negative breast cancer cell lines classified as advanced stage cells. Long-term estrogen depletion assay in MCF-7 cells increased the expression of the 36-38 kDa isoform and reduced the 52-54 kDa isoform, and these cells show an increase in the expression of tumor markers of ER and Her2/neu. MCF-7 LTED cells showed low proliferation and insensitivity to tamoxifen compared to MCF-7 cells in normal conditions. These results support the theory about the relationship of the 36-38 kDa isoform of WT1 and the absence of ER function in advanced breast cancer.

## 1. Introduction

Wilms' tumor gene (*wt1*) consists of 10 exons that encode a zinc finger transcription factor involved in genitourinary development during embryogenesis [1, 2]. Recent studies have described the WT1 protein as an oncogene because, in cancer, it regulates genes responsible for cell growth, apoptosis, and tumoral angiogenesis such as cyclin D1, Bcl-2, Bcl-xL, BFL1, c-myc, and VEGF [3–8]. The WT1 protein contains an N-terminal protein region with a domain involved in transcriptional regulation, self-association, and RNA recognition [9–11] and a C-terminal protein region that contains a DNA/RNA binding domain and four zinc finger domains that can bind to GC-rich sequences [12–14].

The *wt1* gene has two alternative splicing sites, identified as exon 5 or 17AA and another that occurs in exon 9, identified as KTS (Lys-Thr-Ser) [15]. The different isoforms are referred to as (A) the isoform that lacks both the 17 amino acid and KTS inserts, (B) the isoform that contains the 17 amino acid inserts but lacks the KTS insert, (C) the isoform that lacks the 17 amino acid inserts but contains the KTS insert, and (D) the isoform that contains both inserts [16, 17]. Moreover, WTI has three sites of initiation of translation that produce three isoforms of different molecular weights (62-64 kDa, 52-54 kDa, and 36-38 kDa) with different biological properties [18, 19]. The 62-64 kDa WT1 protein isoform is not essential for normal development and reproduction in mice [20], while the 36-38 kDa WT1 protein isoform has more oncogenic potential than the WT1 52-54 kDa protein isoform in leukemia cells [20, 21].

It has been reported that WT1 appears to have a growth regulatory role in many solid cancers including lung [22], colon [23], pancreatic [24], breast [25], and gastric [26], as well as in leukemia [27] and lymphoma [28]. In breast cancer, Silberstein et al. initially considered WT1 to be a tumor suppressor gene, because its overexpression was found in healthy tissue and not in breast cancer tissue [29]; however, subsequent work by Loeb et al. reported that WT1 expression is found in 90% of breast cancer samples [25]. Miyoshi et al. correlated high levels of *wt1* mRNA with a poor prognosis and survival rate in breast cancer patients [30], and Oji et al. demonstrated that the wild-type *wt1* gene plays an important role in the tumorigenesis of breast cancer [31].

Currently, it is not fully known how WT1 interacts with tumor markers used in the characterization of breast cancer, such as the estrogen receptor, progesterone receptor, and Her2/neu. Studies in breast cancer lines have demonstrated that WT1 expression is involved in the modulation of tumor marker expression. Zapata-Benavides et al. found that WT1 protein was decreased by around 60% in MCF-7 cells cultured without estrogen, but WT1 expression was restored after 17*β*-estradiol treatment [32]. Another work related to tumor markers in breast cancer was performed by Tuna et al., where it was observed that the signaling pathway of Her2/neu through Akt regulated levels of WT1 expression, concluding that WT1 protein plays a vital role in the regulation of cell cycle progression and apoptosis [33]. Accordingly, based on these previous studies, we hypothesize that overexpression of *wt1* and a change in isoform allow WT1 to be present at different stages of breast cancer progression.

In this study, we analyzed the expression of WT1 isoforms, ER, and Her2/neu in a long-term estrogen depletion *in vitro* model, to mimic the malignant progression of the breast cancer switch from estrogen-dependent to estrogen-independent growth.

## 2. Methodology

### 2.1. Cell Culture

In the initial assays, MCF-7, BT-474, T47D, SKBR-3, MDA-MB-231, MDA-MB-453, and BT-20 breast cancer cells were obtained from ATCC (American Type Culture Collection, Manassas, VA). All cell lines were grown in DMEM/F12 (Invitrogen, USA) medium supplemented with 10% fetal bovine serum (FBS, Invitrogen, USA) and incubated in 95% air with 5% CO_2_ at 37°C.

### 2.2. Quantitative RT-PCR

The total RNA of tissue was isolated using 1 mL of reagent TRIzol (Life Technologies, Gaithersburg, MD) according to the manufacturer's instructions. The DNAc was performed using 5 *μ*g of total RNA, Superscript II, and oligo (dT_12-18_) under the condition at 42°C for 90 min, followed by heating at 70°C for 10 minutes. Each reaction of PCR real-time was made using 2 *μ*L of DNAc. To Her2/neu was used forward primer 5′-GAGGCACCCAGCTCTTTGA-3′, reverse primer 5′-CGGGTCTCCATTGTCTAGCA-3′, and probe Fam-5′-CCAGGGCATAGTTGTCC-3′NFQ, and to ER was used forward primer CGACATGCTGCTGGCTACA, reverse primer 5′-ACTCCTCTCCCTGCAGATTCAT-3′, and probe Fam-5′-CATGCGGAACCGAGATGA-3′NFQ. The human *β*-actin primer set manufactured by Applied Biosystems was used as the endogenous control. For each reaction, Universal PCR Master Mix was used (Roche Branchburg, New Jersey, USA). The protocol was performed for 40 cycles at 94°C for 30 seconds and 64°C for 30 seconds. The calculation of relative expression was conducted using the Livak method [34].

### 2.3. PCR to Detect Spliced Isoforms of 17AA/KTS

Ratios of isoform spliced of *wt1* gene, 17AA (-)/KTS (-), 17AA (-)/KTS (+), 17AA (+)/KTS (-), and 17AA (+)/KTS (+) to total *wt1* transcripts were obtained by PCR according to Oji et al. [22]. The primer sequences were as follows: F2 5′-GACCTGGAATCAGATGAACTTAG-3 and R2 5-GAGAACTTTCGCTGACAAGTT-3′ to determine the ratio of 17AA (+)/(-) and F3 5′-GTGTGAAACCATTCCAGTGTA-3′ and R3 5′-TTCTGACAACTTGGCCACCG-3′ to determine the ratio of KTS (+)/(-). PCR amplification was performed with 35 cycles at denaturalization 94°C for 60 sec, alignment at 56°C for 60 sec, and extension of 72°C for 90 sec. All PCR products were analyzed on 10% acrylamide gels and visualized with ethidium bromide under UV light.

### 2.4. Western Blot to Detect WT1 Protein Isoforms

Total protein collection was performed using reagent TRIzol according to the manufacturer's instructions. Protein samples (50 *μ*g) were electrophoresed on 12% SDS-polyacrylamide gels and transferred to nitrocellulose membranes. Immunodetection of WT1 protein isoform was performed using a WT1C19 polyclonal antibody (COOH-terminal binding, Santa Cruz Biotechnology); the *β*-actin monoclonal antibody was obtained from Sigma Chemical (St Louis, MO), and anti-mouse and anti-rabbit antibodies conjugated with horseradish peroxidase were purchased from Bio-Rad. Protein bands were visualized by enhanced chemiluminescence using Roche Lumi-Light Western blotting substrate. Subsequently, the bands obtained were analyzed by densitometry using the ImageJ program (https://imagej.nih.gov/ij/download.html). The density of each band was normalized with its respective value of *β*-actin.

### 2.5. Long-Term Estrogen Depletion Assay

MCF-7 cells were grown in DMEM/F12 culture medium supplemented with 10% FBS until 60% confluence. Thereafter, the cells were washed twice with sterile buffer PBS to eliminate the phenol red, and the culture medium was replaced with DMEM/F12 phenol red-free supplemented with 10% charcoal-dextran-treated FBS manufactured by HyClone (Road Logan Utah, USA). The sample was made until reaching 80% of the confluence of cell growth in the flask.

### 2.6. Growth Kinetics

Cell growth was assessed after seeding 2.5 × 10^5^ MCF-7 cells under normal conditions and MCF-7 LTED cells (27 weeks) per well in six-well plates using the respective culture medium. Cells were harvested at 24 and 72 hours and quantified by trypan blue exclusion staining. Each count was done in triplicate and normalized to the initial number of cells.

### 2.7. Assays of MCF-7 LTED Cells Treated with Tamoxifen

MCF-7 cells under normal conditions and MCF-7 LTED cells (27 weeks) were seeded (3 × 10^3^ per well) in a 96-well plate. After 24 hours of incubation, tamoxifen was added at 1.25 *μ*M, 2.5 *μ*M, 5 *μ*M, and 10 *μ*M. Each treatment was carried out in triplicate including control untreated cells. The plates were then incubated for 24 hours, then were analyzed using the MTT assay. The MTT reagent (3-(4,5-dimethylthiazol-2-yl)-2,5-diphenyl tetrazolium bromide) was purchased from Sigma-Aldrich. MTT solution was prepared at 5 *μ*g/mL in PBS buffer; then, 20 *μ*L of the MTT solution was added per well, and then, the plate was incubated at 37°C for 1 hour. Finally, the medium was removed, and 200 *μ*L of DMSO was added to solubilize the formazan salt. The plate was analyzed using a microplate reader (Microplate Autoreader EL311, BioTek Instruments Inc., Winooski, Virginia, USA) at 570 nm to determine the optical density (OD).

### 2.8. Statistical Analysis

The significance of different treatments was determined by analysis of variance and Student's *t*-test. Differences were considered significant at *p* ≤ 0.05 using SPSS software, version 13 (SPSS, Inc., Chicago, IL, USA). All data are expressed as the mean ± the standard error. *p* < 0.05 was considered to indicate a statistically significant difference. To calculate IC_50_, AAT Bioquest, Inc. (2019, September 02) was used, Quest Graph™ IC_50_ Calculator (https://www.aatbio.com/tools/ic50-calculator).

## 3. Results

### 3.1. WT1 Protein Isoform Expression in Breast Cancer Cell Lines

**Figure 1:**
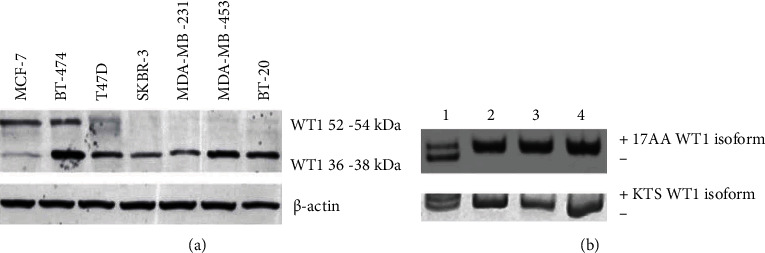
Expression of WT1 isoforms in breast cancer cell lines. (a) The 52-54 kDa and 36-38 kDa WT1 isoforms were analyzed in breast cancer cell lines. The expression of *β*-actin was included as the endogenous control. (b) Analysis of alternative splicing 17AA/KTS in breast cancer cell lines (lane 1: MCF-7; lane 2: BT474; lane 3: SKBR-3; lane 4: MDA-MB-231).

Initially, the characterization of the expression of the WT1 isoforms was carried out in the breast cancer cell lines MCF-7, BT-474, T47D, SKBR-3, MDA-MB-231, MDA-MB-453, and BT-20. WT1 protein expression was detected in all these cell lines, with different isoform patterns. The 52-54 kDa WT1 isoform was expressed only in the estrogen receptor-positive (MCF-7, BT-474, and T47D) but was not expressed in the estrogen receptor-negative cell lines (tumor marker status is shown in Table 1) SKBR-3, MDA-MB-231, MDA-MB-453, and BT-20 (Figure 1(a)). Moreover, the 36-38 kDa WT1 isoform was expressed in all breast cancer cells. To assess 17AA/KTS alternative splicing, four of the seven cell lines were analyzed by conventional RT-PCR. MCF-7 cells expressed four isoforms, and BT-474, SKBR-3, and MDA-MB-231 cells only expressed the 17AA +/KTS - isoform (Figure 1(b)).

### 3.2. MCF-7 Cells in Short-Term Estrogen Depletion Increase the Expression of 52-54 kDa WT1 Protein Isoform

**Figure 2:**
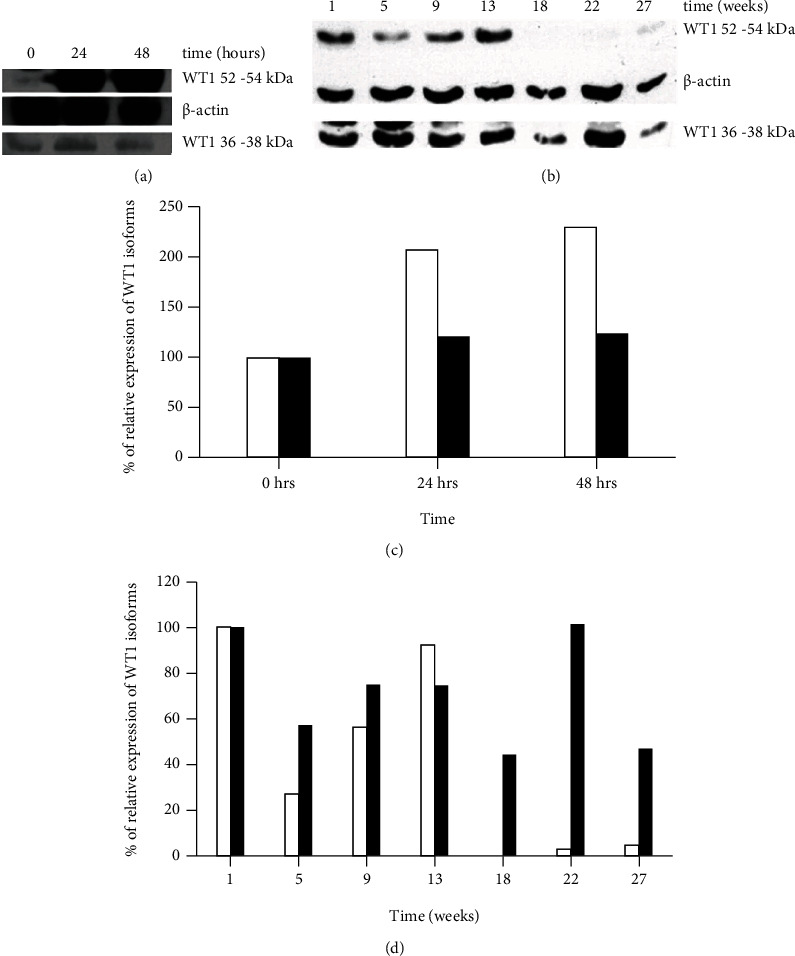
Expression of WT1 in MCF-7 cells cultured with estrogen depletion. (a) The 36-38 kDa and 52-54 kDa WT1 isoform expression in MCF-7 cells in depletion of estrogen in a short term (hours). (b) The 36-38 kDa and 52-54 kDa WT1 isoform expression in MCF-7 cells following the long-term depletion of estrogen (until 27 weeks). In both figures, *β*-actin expression was used as the endogenous control. (c, d) Show the densitometry analysis of the relative expression of WT1 isoforms. The 52-54 kDa isoform is shown in white columns and the 36-38 kDa isoform in black columns.

To analyze the effects of estrogen depletion on WT1 expression, several assays were carried out in MCF-7 estrogen receptor-positive cells. MCF-7 cells cultured under normal conditions were changed to medium under conditions of estrogen depletion for 24 and 48 hours; then, western blotting was performed. The expression of the WT1 protein isoform of 36-38 kDa was not affected considerably; this expression showed an increase of 22% and 25% at 24 and 48 hours, respectively, but the expression of the WT1 protein isoform of 52-54 kDa showed a considerable increase of 108% and 131% at 24 and 48 hours, respectively (Figures 2(a) and 2(c)).

### 3.3. MCF-7 Cells in Long-Term Estrogen Depletion Decrease Their Expression of the 52-54 kDa and Increase the 36-38 kDa WT1 Protein Isoforms

Subsequently, samples of MCF-7 cells were collected over a period of several weeks of long-term estrogen depletion (MCF-7 LTED cells) and were analyzed to determine the expression of WT1 isoforms by western blot. The expression of the 52-54 kDa WT1 protein isoform showed a slight decrease concerning the first week, and this was present until week 13 with a value of 92%; later, the expression decreases from week 18, and it remains low with 3 and 5% in weeks 22 and 27, respectively; however, the expression of the WT1 protein isoform of 36-38 kDa decreases at week 5 to 57%, and it remains present between 44% and 101% in all samples collected during the rest of the weeks analyzed (Figures 2(b) and 2(d)).

### 3.4. The Low Proliferation Rate of MCF-7 Cells Cultured with Long-Term Estrogen Depletion

**Figure 3:**
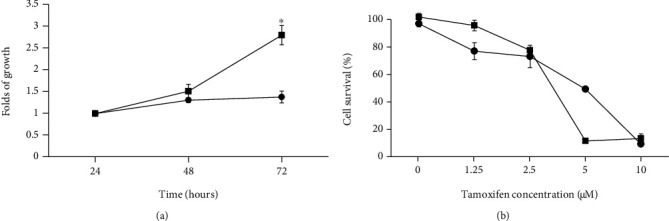
Growth kinetics and effect of tamoxifen on MCF-7 LTED cells. (a) The growth of MCF-7 cells during 27 weeks of estrogen depletion as compared to MCF-7 cells cultured under normal conditions. Cell counts were analyzed at 24 and 48 hours. Each assay was carried out in triplicate with the standard error shown. (b) The sensitivity to tamoxifen was tested using the following concentrations: 1.25 *μ*M, 2.5 *μ*M, 5 *μ*M, and 10 *μ*M in MCF-7 LTED cells (⬤) and MCF-7 cells under normal conditions (⬛). All data are the mean ± SD of three independent experiments (^∗^*p* < 0.05).

Growth kinetic assays were performed to compare MCF-7 cells cultured in medium under normal conditions against MCF-7 LTED cells cultured for 27 weeks in culture medium with estrogen depletion. The assay was performed using the culture medium corresponding to each of the estrogen conditions. MCF-7 LTED cellular proliferation was significantly decreased by 1.4-fold (*p* ≤ 0.05) in comparison with MCF-7 cells cultivated under normal conditions (Figure 3(a)).

### 3.5. Effect of Tamoxifen on MCF-7 LTED Cells

Subsequently, a tamoxifen sensitivity test was performed on MCF-7 LTED cells (27 weeks) compared to MCF-7 cells cultured under normal conditions. MCF-7 LTED cells were less sensitive (IC_50_ = 4.935 mM) to the inhibitory effects of tamoxifen in comparison to MCF-7 cells cultured under normal conditions (IC_50_ = 3.278 mM; *p* ≤ 0.05) (Figure 3(b)). MCF-7 LTED cells were much less sensitive to the antiproliferative effect of tamoxifen.

### 3.6. Analysis of Her2/neu and Estrogen Receptor Expression in MCF-7 LTED Cells

**Figure 4:**
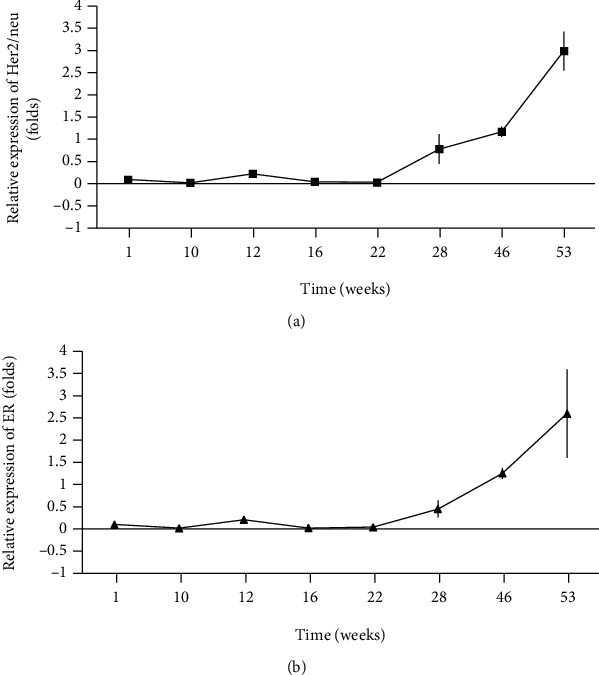
Expression of tumor markers Her2/neu and estrogen receptor (ER) in MCF-7 LTED cells by quantitative RT-PCR. The graph (a) shows the relative expression of Her2/neu (⬛), and graph (b) shows the relative expression of ER (▲). All the experiments were performed in triplicate with *β*-actin as the endogenous control.

To observe the behavior of the molecular markers Her2/neu and ER, a quantitative RT-PCR analysis was performed using MCF-7 LTED cells collected at several time points. The mRNA expression of Her2/neu and ER was significantly increased in MCF-7 LTED cells, starting from week 22 until week 53, with an increase of 2.98-fold (SD ±0.43) and 2.59-fold (SD ±0.99) in the expression of Her2/neu (Figure 4(a)) and ER (Figure 4(b)), respectively.

## 4. Discussion

Breast cancer represents a major health problem worldwide, due to its high incidence and high mortality; it is the second leading cause of cancer death in general and the leading cause of cancer death in women [35]. The molecular classification of breast cancer is performed using the tumor markers progesterone receptor, ER, and Her2/neu. ER and Her2/neu are the most important tumor markers in breast cancer because their presence dictates the type of therapy to be followed and the prognosis of the disease [36, 37]. A critical step in the malignant progression of breast cancer is the switch from estrogen-dependent to estrogen-independent growth [38]. During this change in hormonal dependence, the activity of Her2/neu is increased, leading to a high metastatic potential [39].

High expression of wild-type WT1 in breast cancer has been reported in more than 90% [25, 30], and high levels of WT1 expression have been inversely associated with patient survival. The presence of WT1 is essential for breast cancer cell growth [32]; however, it is still unclear what role it plays during the progression of breast cancer. WT1 can act as a tumor suppressor gene or as an oncogene, possibly due to the presence of various isoforms that have different biological properties [40].

According to the two splicing alternatives, the four isoforms are expressed in several solid cancers, including lung cancer [30], HNSCC [41] sarcoma [42], breast cancer [43], and human primary leukemia [44]. However, the functions of each of the four WT1 isoforms in cancer cells remain unclear [45]. In the present study, we analyzed the WT1 isoforms present in breast cancer cell lines. The presence of the 52-54 kDa WT1 isoform was observed in ER-positive cell lines and not detected in ER-negative cell lines. The 36-38 kDa WT1 isoform was present in all the cell lines analyzed. The biological function of the 36-38 kDa WT1 isoform is not clear yet. The 36-38 kDa WT1 isoform lacks the first 128 amino acids of the N-terminal region, which generates loss of the dimerization domain and a loss of the repression domain. Its presence has been determined in cell lines and specimens of Wilms' tumor where it has been reported that its transactivating function is 1.5-fold higher than the 52-54 kDa WT1 isoform KTS (-). The 36-38 kDa isoform of WT1 may be involved in the progression of the neoplasm due to the lack of the repression domain [19].

To assess the alternative splicing of *wt1*, we observed that MCF-7 cells presented the four isoforms (17AA (+/-)/KTS (+/-)) and the other cell lines only expressed the 17AA (+)/KTS (-)*wt1* isoform. This result agrees with the results reported by Nasomyon et al. where they find that MCF-7 cells express the 4 possible isoforms according to both alternative splicings in WT1 [46]. The 17AA (+) *wt1* isoforms are involved in cell proliferation, apoptosis, and cancer development and protect cells against etoposide-induced apoptosis [47, 48]. Ectopic overexpression of the 17AA (+)/KTS (+) and 17AA (+)/KTS (−) *wt1* isoforms in MCF-7 cells reduces proapoptotic BAK and caspase-7 proteins, as well as p53 mRNA levels [49]. Burwell et al. associated the specific presence of the 17AA (+)/KTS (-) *wt1* isoform with a reduction in cell proliferation and with the appearance of highly organized acinar cellular aggregates; the 17AA (+)/KTS (+) WT1 isoform induced epithelial-mesenchymal transition and the redistribution of E-cadherin [43]. Other work *in vitro* analyzed that the 17AA (-)/KTS (-) WT1 isoform modulates the expression of cytoskeletal regulatory proteins such as *α*-actinin 1, cofilin, and gelsolin, allowing cancer cells to acquire a more aggressive phenotype [50].

Other studies have associated the 17AA (+)/KTS (+) *wt1* isoform with differentiation block, but cell proliferation is induced in 32D cl3 myeloid progenitor cells [51] and normal myeloid cells in response to granulocyte-CSF [52]. Tuna and Itamochi showed that the treatment of MCF-7 cells with insulin-like growth factor I (IGF-I) increases WT1 protein expression by 77%, especially the 17AA (+)/KTS (-) *wt1* isoform [16]. One of the possible hypotheses of the multiple activities of *wt1* isoforms may be due to protein-protein interactions in the 17AA region. Several publications have described interactions of WT1 with proteins such as PAR-4 and p53, so it should be considered in future studies which proteins interact with the various isoforms of WT1 [53–55].

We developed a model to cultivate MCF-7 cells under conditions of estrogen depletion, to mimic the process that occurs naturally with menopause, and the use of aromatase inhibitors as hormonal therapy in breast cancer. In the estrogen depletion trial, an increase in the expression of the 52-54 kDa WT1 isoform was observed at 24 hours, and this expression was observed up to week 13; however, expression disappeared later. On the other hand, the expression of the 36-38 kDa WT1 isoform was not affected in the short-term trial, but the expression was observed from the first week and was fairly constant until week 27. We hypothesize that the increase in the expression of 36-38 kDa WT1 isoform is involved in changes during estrogen depletion, which may be associated with the loss of the repression domain of WT1; this could offset the decrease in proliferation mediated by estrogens since the 36-38 kDa WT1 isoform does not show transcriptional repression [19]. The proliferation of cells cultured with estrogen depletion showed a significant decrease, in addition to presenting a slight insensitivity to the antiproliferative activity of tamoxifen. Zapata-Benavides et al. showed a dose-response effect on the expression of the 52-54 kDa WT1 protein after the administration of 17*β*-estradiol to MCF-7 cells, but the 36-38 kDa WT1 isoform in this work was not analyzed [32]. Artibani et al. in their results found an association between the expression of WT1 with ER-alpha [56]. Nasomyon et al. increased the specific expression of isoforms in MCF-7 cells, and all isoforms showed an increase in the expression of ER-alpha and Her2; however, the 17AA isoforms showed the greatest increases in ER-alpha and Her2 [46]. These results suggest that estrogen stimulation plays a role in modulating WT1 isoforms, thereby affecting cellular behavior.

We then analyzed the quantitative expression of ER and Her2/neu, and in both cases, a gradual increase was observed during MCF-7 estrogen depletion. This coincides with the results of Wang and Wang, who observed estrogen independence in MCF-7 cells through the mitogen-activated protein kinase (MAPK) pathway [38, 40]. In a high passage model of MCF-7 cells (MCF-7H), the estrogen-independent and antiestrogen insensitive growth of MCF7H cells led to high levels of Her2/neu, EGFR, and ER-*α* in breast cancer cells [38], which would explain the increase in ER and Her2/neu transcripts in cells in the context of estrogen depletion. In general, our results show changes in the expression of the different isoforms of WT1 during the adaptation process of MCF-7 cells to estrogen depletion, mainly involving the presence of the 36-38 kDa WT1 isoform and loss of the expression of the 52-54 kDa WT1 isoform. This behavior is related to the tumor status of the analyzed cell lines, as it was observed that only the ER-positive cell lines expressed the 52-54 kDa WT1 isoform; this was absent in the ER-negative cell lines. There are currently few studies where the 36-38 kDa WT1 isoform is analyzed, so it is important to carry out a characterization of the WT1 isoforms present in samples from breast cancer patients with variable tumor marker status. Finally, we conclude that changes in WT1 isoforms during estrogen depletion in MCF-7 cells may play a role in hormone-independent adaptation to tumor growth. With our results, it is very difficult to assign biological properties during this estrogen depletion model; however, it was evident how the isoforms described as truncated by some researchers (36-38 kDa) may be implicit in the aggressiveness of the neoplasia. A recent work shows the transcriptional capacity of the KTS +/- isoforms of WT1, showing that the KTS + isoform has greater transcriptional activation than the KTS - isoform, suggesting the possibility that the insertion of the three amino acids does not inactivate or they reduce the transcriptional capacity, but it can expand their capacity to regulate other promoter regions.

## Figures and Tables

**Table 1:** Tumor marker status of breast cancer cell lines.

	Tumor markers		
Cell line	Estrogen receptor	Progesterone receptor	Her2/neu
MCF-7	+	−	−
BT-474	+	+	+
T47D	+	+	−
SKBR-3	−	−	+
MDA-MB-231	−	−	−
MDA-MB-453	−	−	Low
BT-20	−	−	−

Present (+); absent (-); Her2/neu: human epidermal growth receptor 2. Adapted from [36, 37].

## Data Availability

All the data used to support the findings of this study are included within the article.
